# Lipopolysaccharide Tolerance in Human Primary Monocytes and Polarized Macrophages

**DOI:** 10.3390/ijms241512196

**Published:** 2023-07-30

**Authors:** Hui Li, Annette Breedijk, Nadine Dietrich, Katja Nitschke, Jonas Jarczyk, Philipp Nuhn, Bernhard K. Krämer, Benito A. Yard, Jan Leipe, Sibylle Hauske

**Affiliations:** 1 Fifth Medical Department, University Hospital Mannheim, Heidelberg University, 68167 Mannheim, Germany; hui.li_med@outlook.com (H.L.); annette.breedijk@medma.uni-heidelberg.de (A.B.); nadine.dietrich@medma.uni-heidelberg.de (N.D.); bernhard.kraemer@umm.de (B.K.K.); jan.leipe@umm.de (J.L.);; 2 Department of Urology, University Hospital Mannheim, Heidelberg University, 68167 Mannheim, Germany; katja.nitschke@medma.uni-heidelberg.de (K.N.); jonas.jarczyk@medma.uni-heidelberg.de (J.J.); philipp.nuhn@medma.uni-heidelberg.de (P.N.); 3 European Center for Angioscience (ECAS), Medical Faculty Mannheim, Heidelberg University, 68167 Mannheim, Germany; 4 Center for Innate Immunoscience Mannheim, Heidelberg University, 68167 Mannheim, Germany

**Keywords:** innate immune memory, monocytes, macrophages, LPS tolerance, trained immunity, DMI, TLR agonists, DHA

## Abstract

Innate immune memory allows macrophages to adequately respond to pathogens to which they have been pre-exposed. To what extent different pattern recognition receptors, cytokines and resolution signals influence innate immune memory needs further elucidation. The present study assessed whether lipopolysaccharide (LPS) tolerance in monocytes and macrophages is affected by these factors. Human CD14^+^ cells were isolated from peripheral blood, stimulated by LPS and re-stimulated after 3 days of resting. Hereafter, immune-responsive gene 1 (IRG-1), heme oxygenase 1 (HO-1), tumor necrosis factor α (TNF-α) and interleukin 6 (IL-6) expression were assessed. Our study revealed the following findings: (1) While pre-stimulation with the Toll-like receptor 4 ligand LPS inhibits the induction of IRG-1, TNF-α and IL-6 expression, pre-stimulation with TLR 1/2 ligands only affects cytokine production but not IRG-1 expression upon subsequent TLR4 engagement. (2) Prior TNF-α stimulation does not affect LPS tolerance but rather increases LPS-mediated cytokine expression. (3) Dimethyl itaconate (DMI) inhibits the expression of IRG-1 in a dose-dependent manner but does not affect TNF-α or IL-6 expression. (4) Docosahexaenoic acid (DHA) partly inhibits IRG-1 expression in monocytes but not in M_(IFNγ)_ and M_(IL-4)_ polarized macrophages. LPS tolerance is not affected in these cells by DHA. The data presented in this study partly corroborate and extend previous findings on innate immune memory and warrant further studies on LPS tolerance to gain a better understanding of innate immune memory at the molecular level.

## 1. Introduction

Challenged innate immune cells can display long-term functional changes that increase nonspecific responsiveness to subsequent infections [1]. It is believed that trained immunity or innate immune memory underlies epigenetic reprogramming or rewiring of intracellular metabolic pathways in monocytes, macrophages and other innate cells in response to a variety of stimuli [2,3]. Different stimuli, e.g., lipopolysaccharide (LPS), bacillus Calmette-Guérin or β-glucan, can induce different trained immunity programs [2,4], which seem to influence each other and thus the outcome of subsequent responses to repeated challenges [5]. As such, LPS-stimulated macrophages display adaptive features that result in tolerance upon a secondary LPS challenge; however, tolerance is prevented by prior exposure to *Candida albicans* or the fungal cell wall component β-glucan [6]. While the initiation of Toll-like receptor 4 (TLR4) signaling by LPS has been extensively studied, much less is known about the interaction of β-glucan with its classical receptors Dectin-1 and TLR2. Recent studies have suggested that the induction of trained immunity occurs independently of these receptors [7].

Macrophage stimulation with LPS leads to glycolytic reprogramming to generate ATP and lactate and facilitates the accumulation of tricarboxylic acid (TCA) cycle intermediates, e.g., citrate, succinate, fumarate and malate [8]. Some of these intermediates are diverted from the TCA cycle to support the production of itaconate by aconitate decarboxylase 1 (ACOD1), also known as immune-responsive gene 1 (IRG-1). Itaconate has attracted much attention amongst immunologists due to its broad immunomodulatory properties linked to LPS tolerance [9,10,11,12]. It inhibits succinate dehydrogenase and thereby succinate-mediated inflammatory processes [13,14,15], while at the same time inducing anti-inflammatory proteins such as nuclear factor erythroid 2-related factor 2 (Nrf2) and activating transcription factor 3 (ATF3) [12,15].

Several population-based and experimental studies have suggested that ω-3 fatty acids have beneficial effects in various inflammatory diseases, including atherosclerosis and type II diabetes. Docosahexaenoic acid (DHA) and eicosapentaenoic acid are successively metabolized by 15-lipoxygenase and 5-lipoxygenase to generate resolvins of the D and E series, respectively [16,17,18]. Resolvins are highly active lipids that can attenuate inflammation and promote tissue regeneration. Both are able to reduce prostaglandin synthesis and typical monocyte-/macrophage-derived proinflammatory cytokines, e.g., tumor necrosis factor alpha (TNF-α) or interleukin 6 (IL-6), in response to inflammatory stimuli such as LPS. Although it has been reported that dietary supplementation with ω-3 fatty acids reduced the production of inflammatory cytokines in the peripheral blood mononuclear cells of healthy volunteers [19,20], it is not known whether these compounds also affect the LPS tolerance of monocytes/macrophages. 

The present study sought to investigate to what extent TLR1/2 engagement, inflammatory cytokines (TNF-α) and resolution signals (ω-3 fatty acids /DHA) affect LPS tolerance. In particular, our study addressed the following questions: (1) Does the TLR1/2 ligand Pam3CSK4 cross-tolerize against TLR4 stimulation? (2) Does TNF-α affect LPS tolerance? (3) Is there an association between LPS-mediated IRG-1 induction and LPS tolerance? (4) Do resolution-promoting or anti-inflammatory compounds, e.g., DHA or dimethyl itaconate (DMI), affect LPS tolerance?

## 2. Results

### 2.1. TLR1/2 Ligands but Not TNF-α Partly Tolerize against Subsequent TLR4 Stimulation

**Figure 1:**
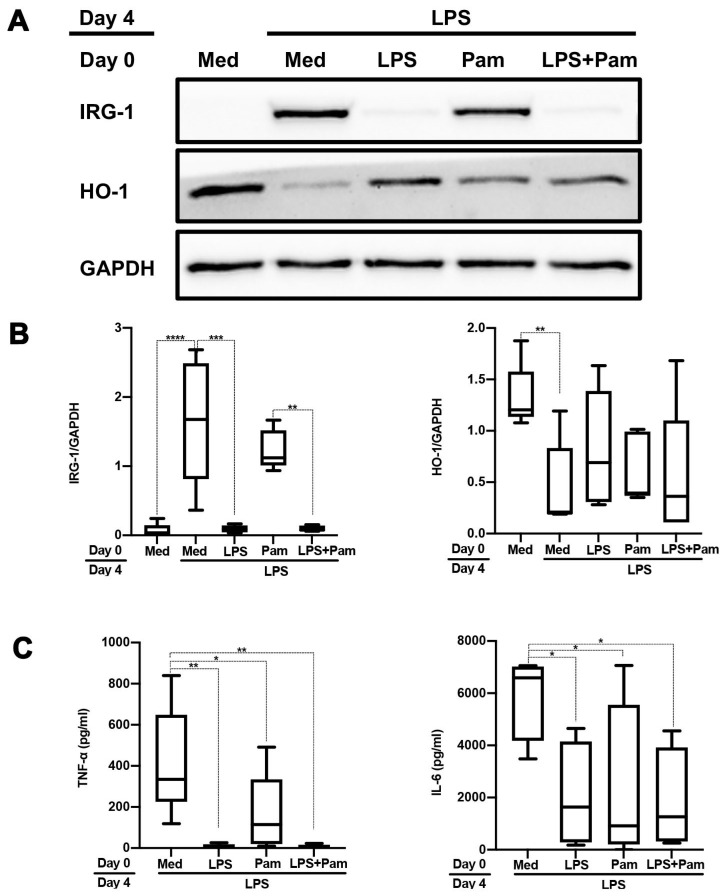
Pam3CSK4 partly cross-tolerizes monocytes. Freshly isolated monocytes were first stimulated for 24 h with pam3CSK4 (50 ng/mL) alone, LPS (500 ng/mL) alone or a combination of both (LPS + Pam) followed by 3 days resting and LPS challenge on day 4. IRG-1 and HO-1 expression were assessed by Western blotting. In (**A**), the results of a representative Western blot are shown. In (**B**), the results of four different experiments were quantified by densitometry and expressed as IRG-1/GAPDH or HO-1/GAPDH ratios. In (**C**), TNF-α and IL-6 production in supernatants were assessed using ELISA. The data in (**B**,**C**) are displayed as box–whisker plots showing the interquartile range (Q1 (25th percentile)–Q3 (75th percentile)), median, minimum and maximum. * *p* < 0.05, ** *p* < 0.01, *** *p* < 0.001, **** *p* < 0.0001, one-way ANOVA with Tukey’s multiple comparisons test.

**Figure 2:**
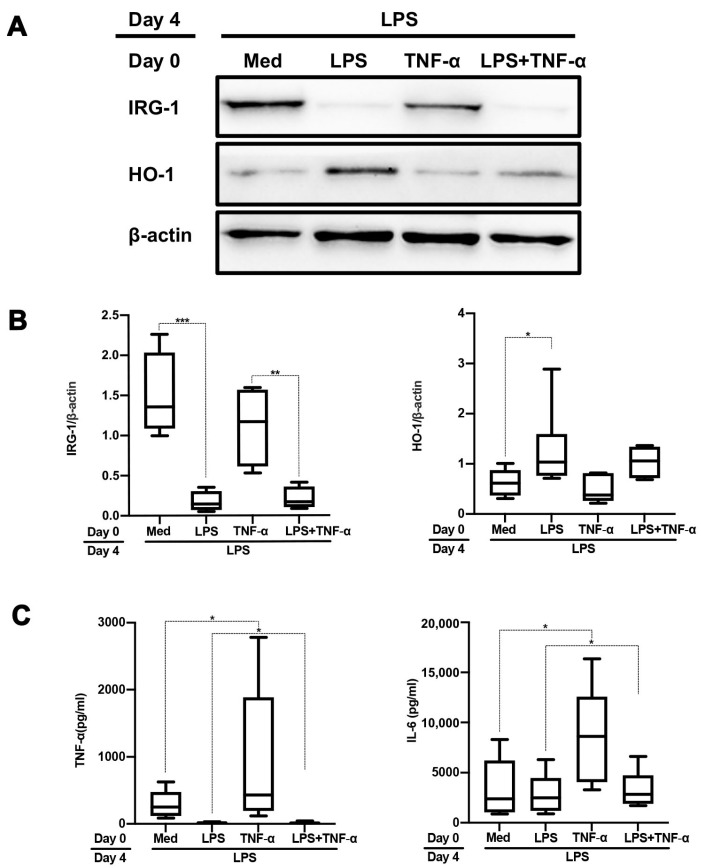
TNF-α does not affect LPS tolerance. Freshly isolated monocytes were first stimulated for 24 h with TNF-α (25 ng/mL) alone, LPS (500 ng/mL) alone or a combination of both (LPS + TNF-α), followed by 3 days resting and LPS challenge on day 4. IRG-1 and HO-1 expression were assessed by Western blotting. In (**A**), the results of a representative Western blot are shown. In (**B**), the results of four different experiments were quantified by densitometry and expressed as IRG-1/β-actin or HO-1/β-actin ratios. In (**C**), TNF-α and IL-6 production in supernatants were assessed using ELISA. The data in (**B**,**C**) are shown as box–whisker plots displayed as the interquartile range (Q1 (25th percentile)–Q3 (75th percentile)), median, minimum and maximum. * *p* < 0.05, ** *p* < 0.01, *** *p* < 0.001, one-way ANOVA with Tukey’s multiple comparisons test.

In vivo and vitro, monocytes and macrophages exposed to LPS become tolerant to a secondary LPS challenge, a process known as “endotoxin or LPS tolerance” [21]. “Cross-tolerance” refers to the ability of an agonist to induce LPS tolerance even though it does not have structural homology with LPS [22]. To address if cross-tolerization occurs when monocytes are stimulated by TLR1/2 ligands, we first isolated monocytes from PBMCs as CD14^+^ cells then stimulated these freshly isolated monocytes with the TLR1/2 ligand Pam3CSK4 (pam) or TLR4 ligand LPS, followed by LPS challenge after 3 days resting in normal culture medium. Monocytes that were stimulated twice with LPS displayed a significantly impaired induction of IRG-1 expression concomitant with a blunted downregulation of HO-1 expression (Figure 1A,B). These cells produced significantly lower amounts of TNF-α and IL-6 as compared to cells that were only stimulated with LPS once. While LPS-mediated IRG-1 induction was not affected by prior pam stimulation, TNF-α and IL-6 production were downregulated, albeit to a lesser extent compared to prior LPS stimulation (Figure 1C). Likewise, LPS-mediated downregulation of HO-1 expression was less pronounced in cells with prior pam stimulation (Figure 1A,B). Prior TNF-α stimulation did not affect LPS-mediated induction of IRG-1 nor did it affect LPS-mediated downregulation of HO-1 expression (Figure 2A,B). Importantly, prior TNF-α stimulation resulted in increased IL-6 and TNF-α production upon a subsequent LPS challenge (Figure 2C).

### 2.2. DMI Dose Dependently Abrogates LPS-mediated IRG-1 Induction

**Figure 3:**
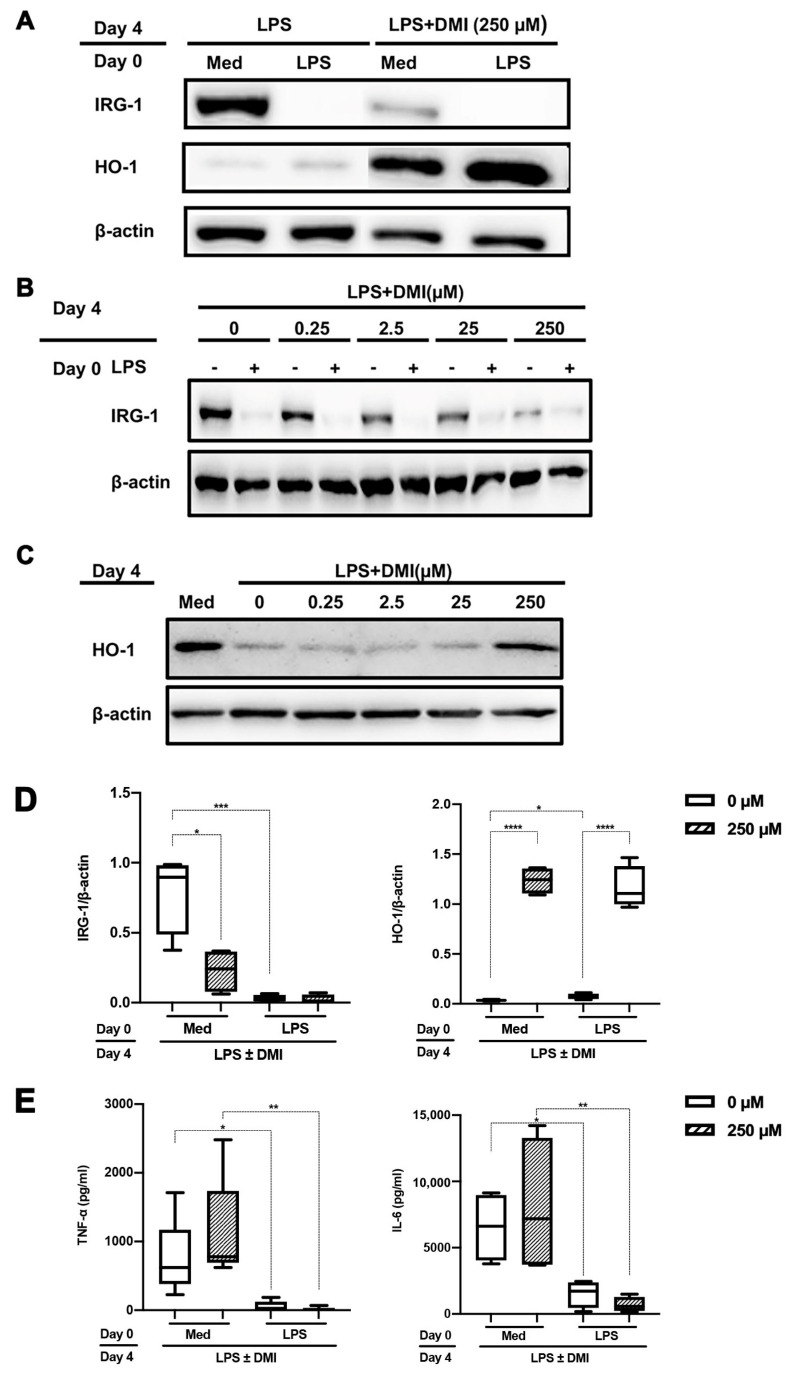
DMI abrogates LPS-mediated IRG-1 induction. Freshly isolated monocytes were first stimulated for 24 h with LPS (500 ng/mL) or left untreated (med). This was followed by 3 days resting and LPS challenge on day 4 in the presence or absence of different DMI concentrations. In (**A**–**C**), representative Western blots are shown for the effect of DMI on IRG-1 and HO-1 expression. In (**D**), the results of four different experiments were quantified by densitometry and expressed as IRG-1/β-actin or HO-1/β-actin ratios. In (**E**), TNF-α and IL-6 production in supernatants were assessed using ELISA. The data in (**D**,**E**) are displayed as box–whisker plots showing the interquartile range (Q1 (25th percentile)–Q3 (75th percentile)), median, minimum and maximum. * *p* < 0.05, ** *p* < 0.01, *** *p* < 0.001, **** *p* < 0.0001, one-way ANOVA with Tukey’s multiple comparisons test.

Since IRG-1 is responsible for endogenous itaconate production, we next assessed the influence of the cell-permeable DMI on LPS tolerance. Monocytes that were challenged twice with LPS did not induce IRG-1 (Figure 3A). Interestingly, DMI dose dependently abrogated LPS-mediated IRG-1 induction in monocytes that were stimulated only once by LPS. At the highest DMI concentration (250 µM), these monocytes displayed a strong HO-1 expression (Figure 3A–D). Moreover, DMI partly rescued HO-1 expression upon LPS stimulation (Figure 3C). In contrast, DMI did not affect IL-6 and TNF-α production nor did it affect LPS tolerance in this setting. Hence, the lack of IRG-1 induction by LPS does not necessarily result in LPS tolerance nor is DMI able to overcome LPS tolerance once monocytes have already been stimulated with LPS (Figure 3E).

### 2.3. DMI Does Not Affect LPS Tolerance in M_(IFNγ)_ and M_(IL-4)_ Polarized Macrophages

**Figure 4:**
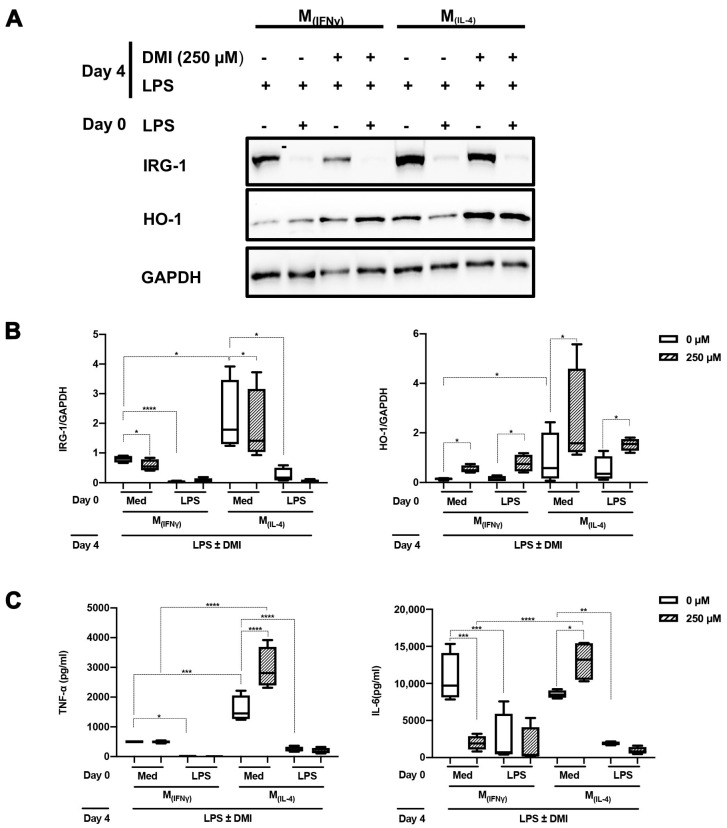
DMI affects LPS tolerance differentially in M_(IFNγ)_ and M_(IL-4)_ polarized macrophages. Peripheral blood monocytes from healthy donors (*n* = 4) were polarized with CSF1 (10 ng/mL) and IFNγ (100 ng/mL) or IL-4 (10 ng/mL) to generate M_(IFNγ)_ and M_(IL-4)_ polarized macrophages, respectively. After 6 days of stimulation, macrophages were considered to be polarized and stimulated with LPS or left untreated for 24 h (day 0). This was followed by 3 days resting and re-stimulation with LPS ± DMI (250 µM) on day 4. IRG-1 and HO-1 expression were assessed by Western blotting. In (**A**), the results of a representative Western blot are shown. In (**B**), the results of four different experiments were quantified by densitometry and expressed as IRG-1/GAPDH or HO-1 GAPDH ratios. In (**C**), TNF-α and IL-6 production in supernatants were assessed using ELISA. The data in (**B**,**C**) are displayed as box–whisker plots showing the interquartile range (Q1 (25th percentile)–Q3 (75th percentile)), median, minimum and maximum. * *p* < 0.05, ** *p* < 0.01, *** *p* < 0.001, **** *p* < 0.0001, one-way ANOVA with Tukey’s multiple comparisons test.

We have recently reported that concurrent stimulation of monocytes with CSF-1 and IFNγ or IL-4 results in polarized macrophages (M_(IFNγ)_ and M_(IL-4)_, respectively) that differ phenotypically and functionally from classically polarized macrophages (M1 and M2) [23]. We therefore assessed to what extent LPS tolerance can be induced in such polarized macrophages and if this was affected by DMI. LPS-mediated induction of IRG-1 was impaired in both M_(IFNγ)_ and M_(IL-4)_ polarized macrophages when stimulated twice. In general, HO-1 expression was higher in the latter type of macrophage. For both types of macrophages, HO-1 expression was significantly higher in cells that were stimulated in the presence of DMI (Figure 4A,B). LPS tolerance was observed with respect to TNF-α and IL-6 production, as reflected by a significantly blunted response upon a second LPS stimulation. In cells that were only stimulated once with LPS, TNF-α and IL-6 production were increased in M_(IL-4)_ but not in M_(IFNγ)_ polarized macrophages when stimulated in the presence of DMI. Under these conditions, IL-6 production was significantly inhibited in M_(IFNγ)_ polarized macrophages (Figure 4C).

### 2.4. DHA Does Not Affect LPS Tolerance

**Figure 5:**
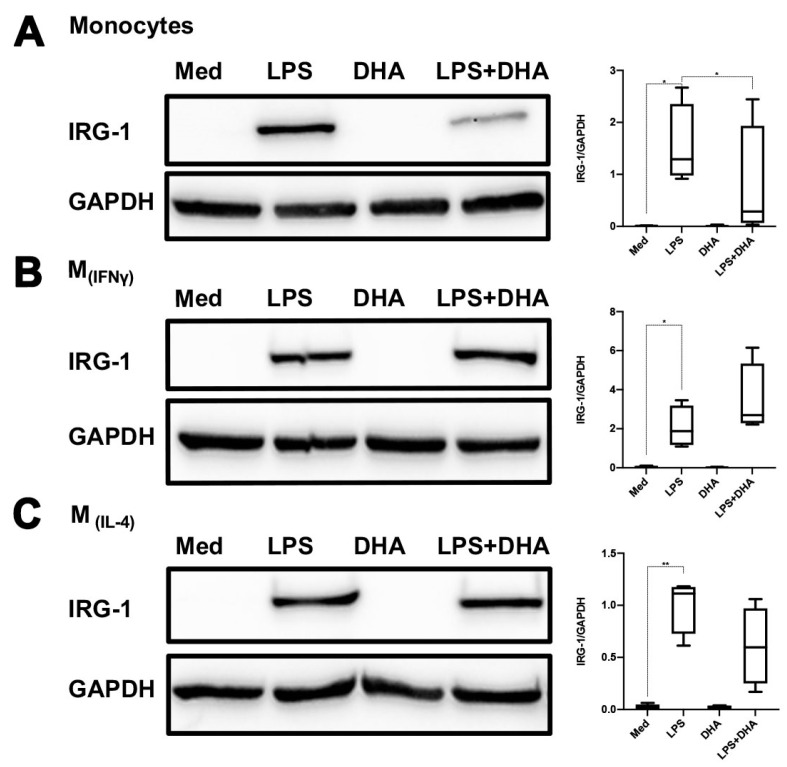

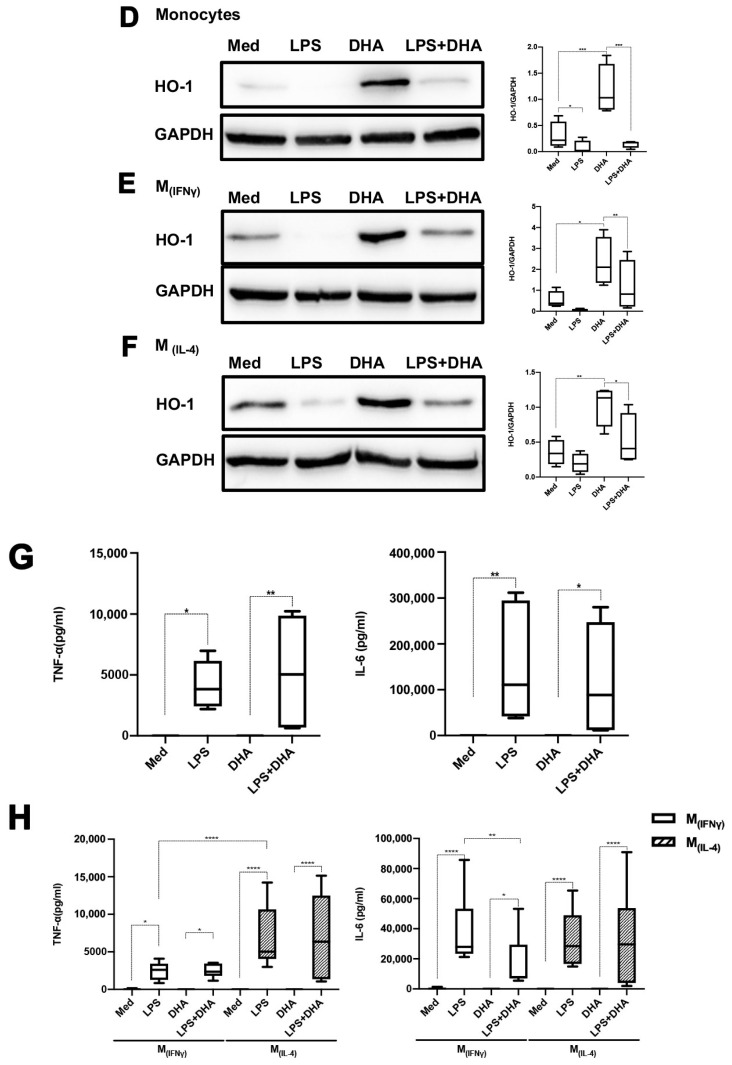
DHA does not affect LPS tolerance. Freshly isolated monocytes and M_(IFNγ)_ and M_(IL-4)_ polarized macrophages were stimulated for 24 h with LPS (500 ng/mL) in the presence or absence of DHA (62.5 µM). Cells that were left untreated (med) or stimulated with DHA alone were also included. IRG-1 (**A**–**C**) and HO-1 (**D**–**F**) expression were assessed by Western blotting. The results of representative Western blots are shown. In (**A**–**F**), the graphs to the right represent densitometric quantifications of four different experiments. In (**G**,**H**), TNF-α and IL-6 production in supernatants of monocytes (**G**) and polarized macrophages (**H**) were assessed using ELISA. The data are displayed as box–whisker plots showing the interquartile range (Q1 (25th percentile)–Q3 (75th percentile)), median, minimum and maximum. * *p* < 0.05, ** *p* < 0.01, *** *p* < 0.001, **** *p* < 0.0001, one-way ANOVA with Tukey’s multiple comparisons test.

**Figure 6:**
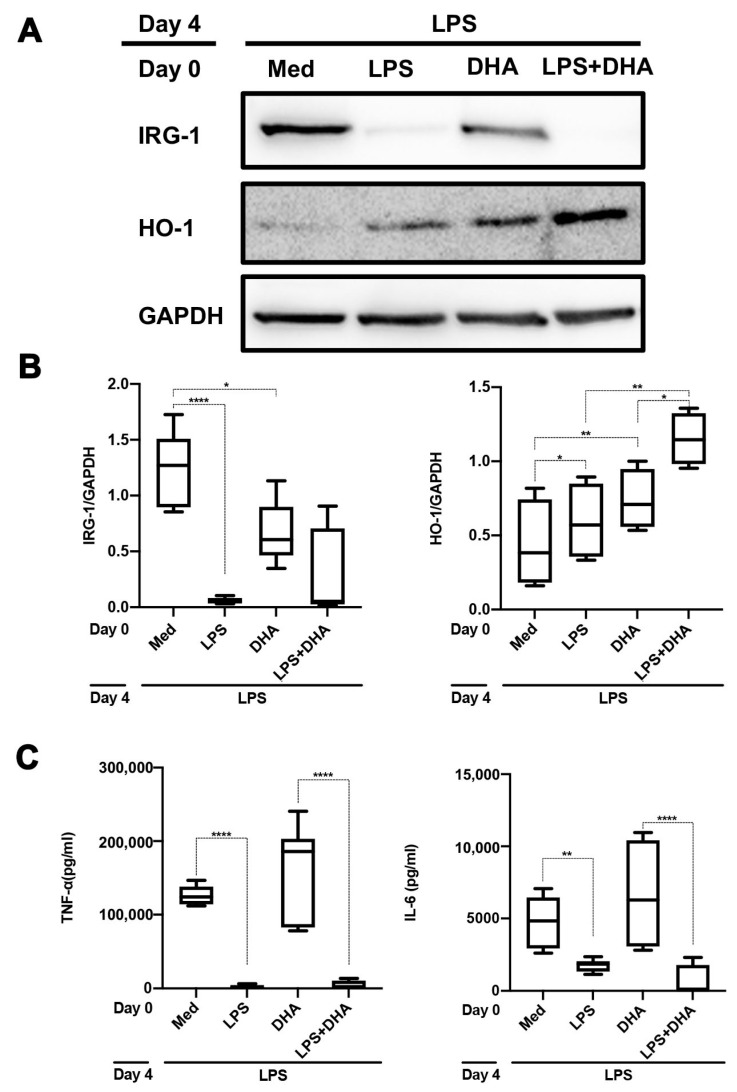

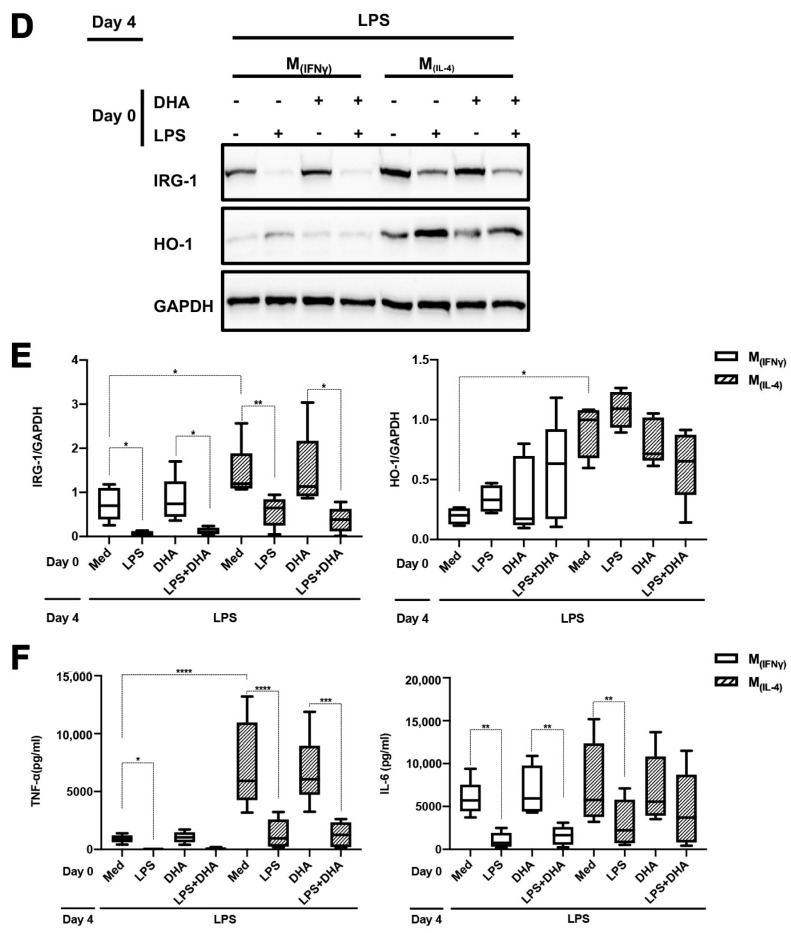
Influence of DHA on LPS tolerance in monocytes and polarized macrophages. Freshly isolated monocytes (**A**–**C**) and M_(IFNγ)_ and M_(IL-4)_ polarized macrophages (**D**–**F**) were stimulated on day 0 for 24 h with LPS (500 ng/mL) ± DHA (62.5 µM). Cells that were left untreated (med) or stimulated with DHA alone were also included. Stimulation was followed by 3 days resting and re-stimulation with LPS on day 4. IRG-1 and HO-1 expression were assessed by Western blotting. In (**A**,**D**), the results of representative Western blots are shown. In (**B**,**E**), the results of four different experiments were quantified by densitometry and expressed as IRG-1/GAPDH or HO-1 GAPDH ratios. In (**C**,**F**), TNF-α and IL-6 production in supernatants were assessed using ELISA. The data in the graphs are displayed as box–whisker plots showing the interquartile range (Q1 (25th percentile)–Q3 (75th percentile)), median, minimum and maximum. * *p* < 0.05, ** *p* < 0.01, *** *p* < 0.001, **** *p* < 0.0001, one-way ANOVA with Tukey’s multiple comparisons test.

It has been reported that DHA mediates anti-inflammatory effects in LPS-stimulated macrophages [24,25]. We therefore addressed whether DHA would interfere with the induction of LPS tolerance in monocytes and polarized M_(IFNγ)_ and M_(IL-4)_ macrophages. We first assessed the effect of DHA on monocytes and polarized macrophages upon a single LPS challenge. As can be seen in Figure 5, DHA significantly blunted IRG-1 induction in monocytes but not in polarized macrophages (Figure 5A–C). HO-1 expression was strongly increased by DHA in all three types of cells, but this did not occur in the presence of LPS (Figure 5D–F). In contrast, LPS-mediated TNF-α and IL-6 production were not significantly influenced by DHA in monocytes, while in polarized M_(IFNγ)_ macrophages, IL-6 production was significantly inhibited by DHA. Polarized M_(IL-4)_ macrophages behaved similarly to monocytes with respect to LPS-mediated cytokine production (Figure 5G,H). When cells were re-stimulated with LPS on day 4, prior DHA treatment also blunted IRG-1 induction only in monocytes but not polarized macrophages (Figure 6A,B vs. Figure 6D,E). Irrespective of DHA treatment, cells that were previously stimulated with LPS remained non-responsive with respect to IRG-1 induction upon a second LPS stimulation. Non-responsive monocytes, i.e., monocytes that were stimulated twice with LPS, also expressed significantly higher amounts of HO-1, particularly when previously exposed to DHA. In polarized M_(IFNγ)_ and M_(IL-4)_ macrophages that were stimulated with LPS on days 0 and 4, a trend for higher HO-1 expression was also noticed as compared to polarized macrophages that were stimulated only on day 4. LPS tolerance with respect to cytokine production was not influenced by DHA nor did DHA influence TNF-α and IL-6 production.

## 3. Discussion

Macrophages and monocytes respond to pathogens in a highly variable manner depending on the nature of the pathogen and the contextual setting of the encounter [24,26]. In this study, we focused on LPS tolerance, a form of trained immunity that aims to protect the host from overproduction of inflammatory mediators [2]. The main findings that our study reveals are the following: (1) the engagement of TLR1/2 ligands prior to TLR4 stimulation partly results in LPS tolerance; (2) TNF-α does not affect LPS tolerance but rather enhances LPS-mediated cytokine production; (3) although DMI dose-dependently inhibits the expression of IRG-1, this does not promote LPS tolerance; (4) DHA affects the LPS-mediated induction of IRG-1 and IL-6 production in monocytes and polarized M_(IFNγ)_ or M_(IL-4)_ macrophages differently but does not affect LPS tolerance.

Sato et al. found that LPS-induced NF-κB activation and TNF-α production were dramatically reduced in murine macrophages pre-treated with the TLR2 agonist macrophage-activating lipopeptide-2 [22]. Also, Medvedev et al. [27] concluded that in murine peritoneal macrophages pre-exposed to IL-1β, the LPS-mediated activation of NF-κB was inhibited in a similar manner as occurs in macrophages pre-exposed to lipoteichoic acid [28]. Our own findings in human primary monocytes are in good agreement with previous findings on cross-tolerance, yet they also suggest that the outcome of this so-called immune paralysis differs between prior TLR4 (LPS) or TLR1/2 (Pam3CSK4) activation followed by secondary TLR4 stimulation. Hence, upon a secondary LPS challenge, TNF-α and IL-6 production were significantly diminished in both LPS and Pam3CSK4 pre-treated monocytes, yet LPS-induced changes in IRG-1 and HO-1 expression were only found for LPS-pre-treated monocytes (Figure 1). Our study did not disclose the mechanism by which Pam3CSK4 pre-treatment inhibits cytokine expression upon a secondary LPS challenge. Published data show that A20, a ubiquitin-editing enzyme, is rapidly induced by Pam3CSK4 [29]. The ability of A20 to terminate TLR-induced immune response [30,31] suggests that Pam3CSK4-mediated induction of A20 might be accountable for the diminished cytokine response. Also, the involvement of glycogen synthase kinase-3, interleukin-1 receptor-associated kinase 4 and miR-132/miR-212 has been suggested to underlie endotoxin tolerance induced by Pam3CSK4 in human primary monocytes [32]. Whether these factors can explain the difference in prior TLR1/2 vs. prior TLR4 engagement on LPS tolerance is the subject of ongoing research.

TNF-α is a crucial cytokine with versatile functions, e.g., promoting inflammation, regulating cell survival and apoptosis [33]. The activation of TNF receptor-1 can initiate cell apoptosis [34] and leads to the activation of distinct transcriptional factors, e.g., NF-κB and c-Jun, which regulate the transcription of survival and pro-inflammatory genes [35]. The possibility that these pro-inflammatory products themselves also take part in LPS tolerance has not been thoroughly studied. Our findings suggest that LPS-mediated TNF-α production does not contribute to LPS tolerance but rather sensitizes monocytes to produce more cytokines upon LPS stimulation (Figure 2).

Itaconate is generated in the mitochondrial matrix via the decarboxylation of cis-aconitate by the enzyme IRG-1. It is a crucial intermediate to block succinate dehydrogenase and executing a variety of anti-inflammatory actions [36,37]. As such, it disables the formation of the of NLRP3 inflammasome [38], allows nuclear Nrf2 translocation [8,11] and inhibits IκBζ in an ATF3-dependent manner [39]. We used the membrane-permeable compound DMI to further study the role of itaconate in immune paralysis. It has been suggested that exogenous DMI is not directly metabolized to itaconate but instead somehow potentiates the effects of LPS activation to increase itaconate biosynthesis [40]. Bambouskova et al. [9] suggested that DMI inhibits IL-6 production in an IκBζ-dependent manner, without influencing TNF-α expression. Throughout the different experiments that were carried out, we observed that LPS-mediated downregulation of HO-1 expression was counteracted by DMI. However, with the exception of M_(IFNγ)_ polarized macrophages, DMI did not inhibit IL-6 production. In fact, DMI increased both TNF-α and IL-6 expression in M_(IL-4)_ polarized macrophages. It remains to be elucidated why IL-6 was only downregulated in M_(IFNγ)_ polarized macrophages. Yet, it should be underscored that most of the experiments performed by Bambouskova et al. [9] involved bone-marrow-derived macrophages, which may be more similar to our M_(IFNγ)_ polarized macrophages and more distinct from monocytes or M_(IL-4)_ polarized macrophages. DMI inhibited LPS-mediated IRG-1 expression in a dose-dependent manner, which corroborates earlier findings in LPS-treated bone-marrow-derived macrophages and Raw 264.7 cells [41]. This suggests that excessive itaconate concentrations act as a negative feedback loop to downregulate IRG-1 expression. The mechanism of this reverse regulation of itaconate on IRG-1 is currently not explained and warrants further studies.

Resolvins and protectins, both produced from ω-3 fatty acids, have anti-inflammatory and inflammation-resolving effects [42,43,44,45,46]. Because of the anti-inflammatory action of DHA, we assessed to what extent DHA pre-treatment would affect innate immune memory in monocytes and polarized macrophages. Similar to reports for a variety of cells [47,48,49], DHA strongly induced the expression of HO-1 in human primary monocytes and polarized macrophages. This response was significantly blunted in the presence of LPS, in line with previous findings on HO-1 downregulation by LPS [50]. We also found that LPS-mediated IRG-1 expression is downregulated in the presence of DHA in freshly isolated primary human monocytes but not in M_(IFNγ)_ and M_(IL-4)_ polarized macrophages. This was even observed when DHA pre-treatment occurred 3 days prior to LPS stimulation (Figure 6). Our study does not support previous findings that DHA inhibits LPS-mediated cytokine production. It should, however, be emphasized that most previous in vitro studies used differentiated macrophages (MØ) and THP-1 cell lines [51,52,53], which may differ significantly from freshly isolated monocytes from PBMCs. Nonetheless, a recent clinical trial in subjects with chronic inflammation revealed that a 10-week intake of DHA resulted in lower TNF-α and IL-6 production in ex vivo-stimulated monocytes [19]. The DHA concentrations used in our study were approximately similar to those reported by others in which anti-inflammatory effects were demonstrated [51,52,53]. Moreover, the DHA concentration used in our study strongly induced the anti-inflammatory protein HO-1, while in the presence of LPS HO-1, induction was significantly blunted. In our view, this supports our finding that DHA does not affect LPS-mediated IL-6 and TNF-α production in monocytes.

### Limitations of This Study

It should be underscored that the intention of this study was to test if pro-inflammatory cytokines (TNF-α) or resolution-promoting factors (DHA) affect LPS tolerance in monocytes and polarized macrophages. As such, it is a purely observational study that was not designed to address the underlying mechanisms of LPS tolerance. Epigenetic profiles and transcription networks have been studied recently and extensively discussed [54,55,56]. The findings that exogenous molecules act as negative feedback of IRG-1 expression are new, yet the mechanism by which this occurs is currently elusive and warrants further confirmatory in vivo studies.

In conclusion, our study demonstrates that monocytes and monocyte-derived macrophages display LPS tolerance upon a second LPS challenge, irrespective of pre-exposure to cytokines, itaconate or resolution-promoting factors. However, monocytes and macrophages differ in LPS-mediated IRG-1 expression when pre-exposed to DHA and also seem to increase cytokine production when pre-exposed to TNF-α or DMI followed by a single LPS stimulation.

## 4. Materials and Methods

### 4.1. Monocyte Isolation and Culture

Human peripheral blood mononuclear cells (PBMCs) from healthy volunteers were obtained from the blood bank (DRK-Blood Bank, Mannheim, Germany) with informed consent. PBMCs were isolated from buffy coats with Ficoll-Paque^TM^ PLUS solution (Merck, Darmstadt, Germany, cat.no.17-1440-03) using density gradient centrifugation. In brief, buffy coats were diluted 1:4 in PBS+ (PBS (Sigma-Aldrich, Schnelldorf, Germany, cat.no.D8537) containing 2.5 mM EDTA (Merck, Darmstadt, Germany, cat.no.E7889)) and layered on 10 mL of Ficoll-Paque^TM^ PLUS solution followed by centrifugation for 30 min at 400 g. Then, the interphase was pipetted into a new tube and washed several times with cold PBS to remove residual platelets. PBMCs were resuspended in PBS++ (PBS+ containing 0.5% BSA (Miltenyi Biotec, Bergisch Gladbach, Germany, cat.no.130-091-376)) in a final concentration of 1 × 10^8^ cells/mL. Monocytes were isolated from PBMCs by positive selection using magnetic CD14 Nanobeads (Biolegend, Amsterdam, the Netherlands, cat.no. 480093), LS columns (Miltenyi Biotec, Bergisch Gladbach, Germany, cat.no.130-042-401) and a Midi-Magnetic Cell Isolation Separator (Miltenyi Biotec, Bergisch Gladbach, Germany). The cells were cultured in 6-well flat-bottom plates (1–2 × 10^6^ cells/well) in RPMI 1640 medium supplemented with 10% fetal bovine serum and 0.5 units/mL of penicillin–streptomycin (all from Thermo Fisher, Darmstadt, Germany, cat.no. 61870-010; 10500-064; 09-757F, respectively) at 37 °C and 5% CO_2_.

### 4.2. Macrophage Polarization

Human CD14^+^ cells were polarized to M_(IFNγ)_ and M_(IL-4)_ as previously described [23]. In brief, for M_(IFNγ)_ and M_(IL-4)_ polarization, CD14^+^ cells were concurrently stimulated for 6 days with 10 ng/mL of colony-stimulating factor 1 (CSF-1) and 100 ng/mL of IFN-γ or 10 ng/mL IL-4 (all from PeproTech, Hamburg, Germany, cat.no.300-25; 300-2; 200-04, respectively).

### 4.3. Innate Immune Memory Assay

Monocytes and polarized M_(IFNγ)_ and M_(IL-4)_ macrophages were pre-treated or left untreated for 24 h with 500 ng/mL LPS (Sigma-Aldrich, Schnelldorf, Germany, cat.no.L2880) alone, 50 ng/mL Pam3CSK4 (InvivoGen^@^, San Diego, CA, USA, cat.no.tlrl-pms) or 25 ng/mL TNF-alpha (PeproTech, Hamburg, Germany, cat.no.300-01A) alone or in combination with LPS. The concentration and timing used for Pam3CSK4 was based on pilot dose–response experiments. In some experiments, cells were treated with 62.5 μM DHA (Merck, Darmstadt, Germany) in the presence or absence of LPS (500 ng/mL). Pre-treatment was followed by 3 days of resting in fresh culture medium without stimuli. On day 4, the cells were re-stimulated for 18 h with LPS (500 ng/mL) either in combination or not with dimethyl itaconate (DMI, 0.25 to 250 µM depending on the specific experiment) (Sigma-Aldrich, Schnelldorf, Germany, cat.no.592498). Then, supernatants were collected, and cells were lysed for ELISA and Western blot analysis. FACS staining using 7-AAD and Annexin V was performed to exclude cell death as a cause for non-responsiveness.

### 4.4. Western Blot Analysis

Cells were resuspended in lysis buffer (10 mM Tris pH 7.4, 150 mM NaCl, 5 mM EDTA, 1% Triton X-100, 0.5% Na-deoxycholate, 0.1M DTT) containing phosphatase and protease inhibitor. Protein concentrations were measured using a Coomassie (Bradford) Protein Assay Kit (Thermo Fisher, Darmstadt, Germany, cat.no.23200) according to the manufacturer’s instructions. For each sample, 12 µg of protein was denatured for 10 min at 95 °C in Laemmli sample buffer (Bio-Rad, Feldkirchen Germany, cat.no.161-0147) before loading on a 10% SDS-PAGE gel followed by semi-dry blotting onto polyvinylidene fluoride membranes using a Trans-Blot TurboTransfer System(Bio-rad, Feldkirchen Germany, cat.no.1704150). The membranes were blocked with TBS containing 0.1% Tween-20 and 5% non-fat dry milk at room temperature for 1 h and incubated overnight at 4 °C with specific primary antibodies directed against IRG-1 (Abcam, Cambridge, UK, cat.no.222411), heme oxygenase (HO)-1 (ENZO, cat.no.ADI-SPA-895), GAPDH (Santa Cruz, Heidelberg, Germany, cat.no.ADI-SPA-895;sc-47724) or β-Actin (Sigma-Aldrich, Schnelldorf, Germany, cat.no.A5441). The membranes were then extensively washed and incubated with appropriate horseradish-peroxidase-conjugated secondary antibodies. Immune-reactive bands were detected with chemiluminescence using Western Lightning Plus ECL and the Fusion SL Vilber Lourmat Imaging system (Peqlab, Erlangen, Germany, cat.no. NEL104001EA).

### 4.5. Cytokine Measurements

Concentrations of cytokines TNF-α and IL-6 in supernatants of monocytes and polarized macrophages were measured using ELISA according to the manufacturer’s instructions (Biolegend, Amsterdam, The Netherlands, cat.no.DTA00C and D6050, respectively). All samples were analyzed in triplicate.

### 4.6. Statistics

All data were statistically analyzed with GraphPad Prism 9.0 (La Jolla, CA, USA) and depicted with whisker boxplots showing the interquartile range Q1 (25th percentile)–Q3 (75th percentile), median, minimum and maximum. Ordinary one-way ANOVA was adopted to compare the differences between groups. *p* value < 0.05 was considered statistically significant (* *p* < 0.05; ** *p* < 0.01; *** *p* < 0.001; **** *p* < 0.0001).

## Data Availability

All data presented in this study are contained in this article.

## Abbreviations

ATF3	Activating transcriptional factor 3
BCG	Bacille Calmette-Guérin
DHA	Docosahexaenoic acid
DMI	Dimethyl itaconate
HO-1	Heme oxygenase 1
IFNγ	Interferon-gamma
IL-6	Interleukin 6
IRG-1	Immune-responsive gene 1
IκBζ	Inhibitor of Kappa B zeta
LPS	Lipopolysaccharide
LTA	Lipoteichoic acid
M-CSF	Monocyte-colony stimulating factors
NF-κB	Nuclear factor kappa-light-chain-enhancer of activated B cells
Nrf2	Nuclear factor erythroid 2-related factor 2
PBMCs	Peripheral blood mononuclear cells
SDH	Succinate dehydrogenase
TCA	Tricarboxylic acid
TLR	Toll-like receptor
TNF	Tumor necrosis factor
